# Cortisol Modulation by Ayahuasca in Patients With Treatment Resistant Depression and Healthy Controls

**DOI:** 10.3389/fpsyt.2018.00185

**Published:** 2018-05-08

**Authors:** Ana C. de Menezes Galvão, Raíssa N. de Almeida, Erick A. dos Santos Silva, Fúlvio A. M. Freire, Fernanda Palhano-Fontes, Heloisa Onias, Emerson Arcoverde, João P. Maia-de-Oliveira, Dráulio B. de Araújo, Bruno Lobão-Soares, Nicole L. Galvão-Coelho

**Affiliations:** ^1^Laboratory of Hormone Measurement, Department of Physiology, Federal University of Rio Grande do Norte, Natal, Brazil; ^2^Postgraduate Program in Psychobiology, Federal University of Rio Grande do Norte, Natal, Brazil; ^3^Brain Institute, Federal University of Rio Grande do Norte, Natal, Brazil; ^4^Onofre Lopes University Hospital, Federal University of Rio Grande do Norte, Natal, Brazil; ^5^Department of Clinical Medicine, Federal University of Rio Grande do Norte, Natal, Brazil; ^6^National Institute of Science and Technology in Translational Medicine, Natal, Brazil; ^7^Department of Biophysics and Pharmacology, Federal University of Rio Grande do Norte, Natal, Brazil

**Keywords:** ayahuasca, awakening salivary cortisol, plasma cortisol, treatment-resistant depression, hypocortisolemia

## Abstract

Major depression is a highly prevalent mood disorder, affecting about 350 million people, and around 30% of the patients are resistant to currently available antidepressant medications. Recent evidence from a randomized controlled trial (RCT) supports the rapid antidepressant effects of the psychedelic ayahuasca in treatment-resistant depression. The aim of this study was to explore the effect of ayahuasca on plasma cortisol and awakening salivary cortisol response, in the same group of treatment-resistant patients (MD) and in healthy volunteers (C). Subjects received a single dose of ayahuasca or placebo (dosing session), and both plasma and awakening salivary cortisol response were measured at baseline (before dosing session) and 48 h after the dosing session. Baseline assessment (D0) showed blunted awakening salivary cortisol response and hypocortisolemia in patients, with respect to healthy controls. Salivary cortisol was also measured during dosing session, and we observed higher increases for both C and MD that ingested ayahuasca than placebo. After 48 h from the dosing session with ayahuasca, patients' awakening salivary cortisol response is similar to the ones detected in controls. No significant changes in plasma cortisol levels were observed 48 h after the sessions. Therefore, these findings point to new evidence on the modulation of salivary cortisol levels as a result of an ayahuasca session, both in healthy and depressive volunteers. Considering that cortisol acts in regulation of distinct physiological pathways, emotional and cognitive processes, it is assumed to be critically involved to the etiology of depression and its regulation seems to be important for the treatment and remission of major depression, ayahuasca use as antidepressant should be further investigated. Moreover, this study highlights the importance of psychedelics in the treatment of human mental disorders.

## Introduction

Major depression is a highly prevalent mood disorder, affecting about 350 million people worldwide (1). It is more prevalent in women than men and has a huge impact on general health of the patients (1, 2).

Major depressive disorder has been closely associated with deregulations of the hypothalamic-pituitary-adrenal (HPA) axis, both at rest and in response to stress (3–5). Some studies report changes in cortisol response that occur soon after awakening (6). Most often reported, cortisol awakening response is increased in patients with major depression, suggesting hyperactivity of the HPA axis (7). However, there is increasing evidence of hypocortisolism in patients with depression, which has been interpreted as an indication of HPA axis fatigueness in response to recurrent depressive episodes (8, 9). Such discrepancies can be attributed to a number of factors including subtypes of depression, depression severity, sex, duration of illness and socioeconomic status (10–13).

Cortisol assessments have also served as an important biomarker of treatment response. For instance, patients with depression who responded to an 8-week treatment with fluoxetine, a selective serotonin reuptake inhibitor, presented decreased levels of cortisol (14).

Most often, patients are treated with antidepressant medications that at present take about 2 weeks for the beginning of their therapeutic effects (15–18). Recently, however, psychedelics have been emerging as a promising fast-acting antidepressant (19–23). A recent open label trial in treatment-resistant depression observed a reduction of up to 87% in depression severity, already at 24 h after a single dosing session with ayahuasca (23, 24). Ayahuasca was originally used for medicinal purposes by indigenous populations from Brazil, Ecuador, Peru, and Colombia, and later its ritualistic use became more popular by its presence in ceremonies of different syncretic churches in Brazil, which is currently spreading to other parts of the world (25).

Ayahuasca is a decoction of two plants: *Psychotria viridis* and *Banisteriopses caapi* (26). *P. viridis* contains the psychedelic tryptamine N,N-dimethyltryptamine (N,N-DMT), whose action is mediated by serotonin (5-HT2A) and sigma-1 receptors (27–30). *B. caapi* contains β-carbolines (harmaline, harmine and tetrahydroharmine), which work as indirect monoaminergic agonist due to the inhibition of monoamine oxidase isoenzyme (MAO) (31, 32). Regular ayahuasca users in religious contexts have shown low level of psychopathologies (33–35), low scores on the state scales related to panic and hopelessness (36), as well as good performances in cognitive neuropsychological tests (37, 38). Moreover, this brew does not exhibit dose tolerance, i.e., the decrease of the effect of a drug or medication by excessive or frequent exposure of the patient to its active principle, and is not addictive (39–41).

Considering that the main neurobiological actions of ayahuasca are strongly related to key physiological systems altered in major depression, i.e., was observed that ayahuasca increased cortisol levels 2 h after its ingestion in healthy subjects (42) and taking into account the low incidence of mental disorders in regular ayahuasca users in religious context and previous results from open label trial (23), we recently conducted a randomized controlled trial (RCT) with ayahuasca in patients with treatment-resistant depression. Our results suggest significant and rapid reduction in depressive symptom after a single ayahuasca session, when compared to placebo (43). Herein, we explored the effects of ayahuasca on the awakening salivary cortisol response and plasma cortisol, in patients with treatment-resistant depression and in healthy individuals.

Our hypotheses are that patients and controls will show different levels of plasma cortisol and awakening salivary cortisol response, at baseline. Furthermore, we expect that the cortisol levels in the group of patients will be correlated with the severity and/or duration of disease. Moreover, we expect that ayahuasca, but not placebo, will increase cortisol levels acutely, after the dosing session and after 48 h of its ingestion, in patients and controls. Changes in cortisol levels will be correlated with the observed improvement in the symptoms of depression.

## Methods

This is a randomized double-blinded placebo-controlled trial using a parallel arm design. Patients were referred from psychiatric units of the Onofre Lopes University Hospital (HUOL), in Natal/RN, Brazil, and through media and internet advertisements. All procedures took place at the HUOL. The authors assert that all procedures contributing to this work comply with the ethical standards of the relevant national and institutional committees on human experimentation and with the Helsinki Declaration of 1975, as revised in 2008. The study was approved by the Research Ethics Committee of the University Hospital (# 579.479), and all subjects provided written informed consent prior to participation. This study is registered at http://clinicaltrials.gov (NCT02914769).

### Volunteers

Seventy-one volunteers participated in the study: 43 healthy controls (C) (19 men and 24 women) without history or diagnosis of major illness or psychiatric disorders, and 28 patients with treatment resistant depression (MD), (7 men and 21 women), defined as those patients with inadequate responses to at least two antidepressants from different classes (44). Patients were screened for exclusion due to previous experience with ayahuasca, current clinical medical disease, pregnancy, current or previous history of neurological disorders, history of schizophrenia, or bipolar affective disorder, history of mania or hypomania, use of substances of abuse, and suicidal risk. Selected patients were in a current moderate to severe depressive episode at screening by the Hamilton Depression Rating Scale (HAM-D≥17). All patients were not using any antidepressant medication during the trial, they were in pharmacological wash-out phase of approximately 2 weeks, however they all were under regular use of benzodiazepines.

Volunteers from both groups (healthy and patients) were randomly assigned (1:1) to receive ayahuasca or placebo using 10-gauge blocks. Half of the patients and half of the controls received ayahuasca while the other half received placebo. All investigators and patients were blinded to the intervention assignment. The Montgomery-Åsberg Depression Rating Scale (MADRS) monitored depressive symptoms at admission of patient to the psychiatry division of HUOL (D-1), and 2 days after the dosing session (D2).

### Ayahuasca and placebo

The substance used as placebo was not psychoactive, but induced a light gastrointestinal discomfort, and simulated some organoleptic properties of ayahuasca. It is a brown liquid with a bitter and sour taste, containing water, yeast, citric acid, zinc sulfate, and a caramel dye.

A single batch of ayahuasca was used throughout the study. It was prepared and supplied free of charge by a branch of the Barquinha church, based in the city of Ji-Paraná, Brazil. The alkaloid concentrations in the ayahuasca batch were analyzed by mass spectroscopy twice during the trial. On average, the ayahuasca contained (mean ± SD): 0.36 ± 0.01 mg/mL of N,N-DMT, 1.86 ± 0.11 mg/mL of harmine, 0.24 ± 0.03 mg/mL of harmaline and 1.20 ± 0.05 mg/mL of tetrahydroharmine (THH).

### Salivary cortisol

Saliva was collected using a specific cotton stick called Salivette (Sarstedt, Germany). Volunteers were instructed to place the cotton in their mouth without touching it and masticating it for a period of about 1–2 min. Before and during collection, subjects remained at rest and no liquid or food were allowed.

Saliva samples were stored at −80°C in the Laboratory of Hormonal Measures of Federal University of Rio Grande do Norte (UFRN) and the salivary cortisol was measured using the ELISA DGR – SLV 4635 kit (DGR International, Inc, Germany).

### Plasma cortisol

Blood samples were collected for total plasma cortisol (PC) assessment. All volunteers were at fast and at complete rest for 45 min prior to blood sampling. Samples were stored at −80°C in the Laboratory of Hormonal Measures (UFRN). Total plasma cortisol was measured by ELISA using the DGR-SLV 1887 kit (DGR International, Inc, Germany).

### Experimental procedure

**Figure 1:**
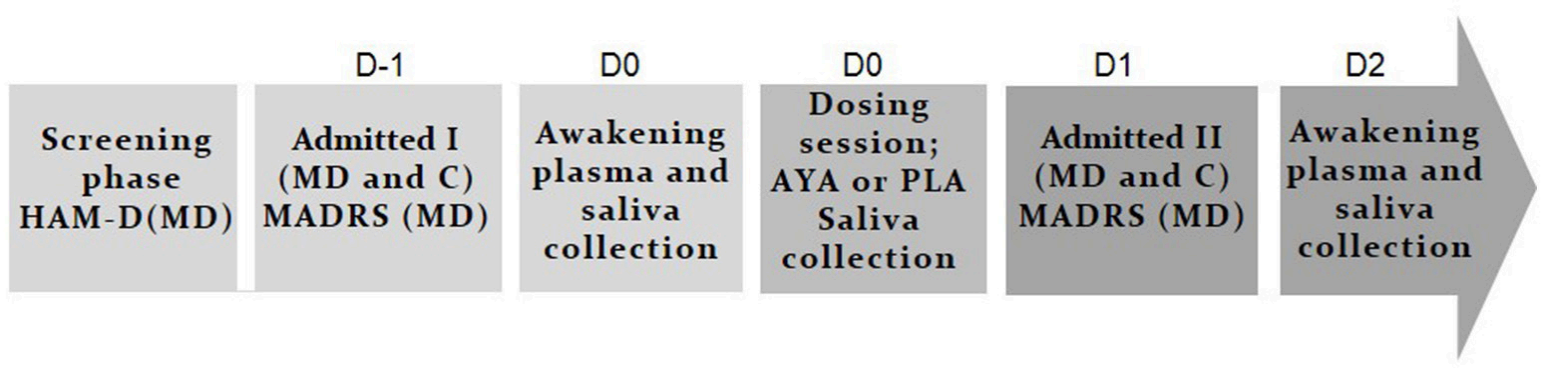
Experimental design. In baseline (color: light gray) patients with treatment resistant depression (MD: 7 men and 21 women) and healthy controls (C: 19 men and 24 women) were selected in the screening phase. MD were in a pharmacological wash-out phase (±2 weeks) and were screening by the Hamilton Depression Rating Scale (HAM-D). In D-1 all volunteers slept at the hospital (admitted I) and Montgomery-Åsberg Depression Rating Scale (MADRS) scale of depression was applied for DM. At D0, plasma, and saliva collection at awake were performed for cortisol dosage. Around 10:00 a.m. happened dosing session with ayahuasca (AYA) or placebo (PLA) (color: gray). Acute salivary cortisol changes (%) were assessed during dosing session. At D1 all volunteers slept again at the hospital (admitted II) and on D2 (48 h after dosing session) plasma and saliva collection at awake were performed and MADRS was applied (color: dark gray).

Figure 1 shows the experimental design of the study. After screening phase, volunteers were admitted to the psychiatry division of HUOL (admitted I) one night before dosing session (D-1), when the MADRS scale of depression was applied for patients. The volunteers slept in the hospital. At 6:00 a.m. the next day (D0) saliva samples were collected for measuring awakening salivary cortisol and at 7:00 a.m. the blood samples were collected for PC assessment. Awakening salivary cortisol response assessments consisted of 3 saliva samples collected at (i) awakening (±5 min), (ii) +30 min, and (iii) +45 min.

Dosing session started around 10:00 a.m. (D0). Volunteers received a single dose of 1 ml/kg of ayahuasca (AYA) adjusted to contain 0.36 mg/kg of N,N-DMT, or 1 ml/kg of placebo (PLA). During the entire session subjects were asked to remain quiet with their eyes closed while concentrating on their body, thoughts and emotions. They could listen to a pre-defined music playlist, if they wish. Volunteers were supported by at least two researchers offering assistance when needed. Acute salivary cortisol changes (%) were assessed during dosing session at two instants: (i) immediately before dosing, and (ii) +1 h 40 min after the ingestion of placebo or ayahuasca.

On the following day (D1), volunteers slept again in the hospital (admitted II). At 6:00 a.m. the next day (D2), 48 h after dosing session, saliva samples were collected for measuring awakening salivary cortisol, at 7:00 a.m. the blood samples were collected for PC assessment and MADRS scale of depression was applied for patients.

### Statistical analysis

Statistical analysis was conducted in Statistic 12.5 and the level of significance was set at p < 0.05 for all tests. Graphics were built in R 3.4.1 (RStudio).

The area under the curve (AUC) was calculated from the 3 points of salivary cortisol at waking time. Both salivary and plasma cortisol levels were normalized by the logarithm to use parametric tests.

A parametric test of Analysis of Covariance (ANCOVA) was used to analyze differences between groups (healthy and patients) at baseline, for both salivary (AUC of awakening salivary cortisol) and plasma cortisol. Sex was inserted as co-variable.

At baseline, *Spearman* correlations were calculated between the changes in clinical outcome scales (HAM-D and MADRS), the duration of depression and both plasma cortisol and AUC of awakening salivary cortisol.

General Linear Models (GLM) and Fisher *post-hoc* tests were used to evaluate interaction between changes of AUC of awakening salivary cortisol response at D0 and D2, considered as dependent quantitative variable. Sex (men and women), groups (healthy volunteers and patients) and treatment (AYA or PLA) were considered as independent qualitative variables. General Linear Models (GLM) and Fisher *post-hoc* tests were also used for plasma cortisol levels. In this case, sex was not used as independent variable, due to the small number of male patients who received placebo (*n* = 2).

Acute salivary cortisol changes (%) during the dosing session were evaluated 1 h 40 into the dosing session and assessed by Mann-Whitney test.

Furthermore, *Spearman* correlations were calculated between acute salivary cortisol changes (%), during the dosing session, plasma cortisol (at D2), AUC of awakening salivary cortisol (at D2) and with MADRS scores (at D2).

## Results

Socio-demographic characteristics of healthy controls (C), and patients with major depression (MD) are summarized in Table 1. All volunteers (*n* = 71; MD = 28, C = 43) are Brazilian, adults [MD = 41.54 ± 11.55, C = 31.21 ± 9.87 years, *t*_(69)_ = 4.03 *p* < 0.0001]. From January 2014 to June 2016, 218 patients were screened for eligibility, and 35 met criteria for the trial. On average, patients presented 11.03 ± 9.70 years of depressive symptoms and met criteria for moderate-to-severe depression (HAM-D = 21.83 ± 5.35). They were treated previously with 3.86 ± 1.66 different types of antidepressants and two patients used electroconvulsive therapy as treatment. The majority of patients presented comorbidity, such as personality disorder (76%) and anxiety disorder (31%).

**Table 1:** Socio-demographic characteristics of 71 volunteers who participated in the study: 43 healthy controls (19 men and 24 women) without history or diagnosis of major illness or psychiatric disorders, and 28 patients with treatment-resistant depression (7 men and 21 women).

	**Controls**	**Patients**	**Statistical analysis**
Participants, *n*	43	28	
Age (years)	31.21 ± 9.87	41.54 ± 11.55	*t*(69) = 4.03 *p* < 0.0001
Gender (M/F)	19/24	7/21	*X*^2^(1) = 2.69 *p* = 0.10
Unemployed (%)	5/43 (12)	15/28 (54)	*X*^2^(1) = 14.74 *p* < 0.0001
Household income			*X*^2^(3) = 14.03 *p* = 0.003
<5 wages (%)	19/86 (71)	22/56 87	
6–10 wages (%)	14/43(7)	2/28 (6.6)	
11 or more wages (%)	10/43 (21)	4/28 (6.6)	
Education			*X*^2^(3) = 19.88 *p* = 0.0002
Up to 8 years, *n* (%)	3/43 (7)	11/28 (39)	
9–11 years, *n* (%)	4/43 (9)	8/28 (29)	
12–16 years, *n* (%)	18/43(42)	4/28 (14)	
17 or more years, *n* (%)	18/43 (42)	5/28 (18)	

Patients showed significant lower socioeconomic status backgrounds than healthy volunteers: large part of the MD group was from low socioeconomic status, unemployed [MD = 54%, C = 12%, *X*^2^(1) = 4.74, *p* < 0.0001], earning up to 5 minimum wages [MD = 87%, C = 71%, *X*^2^(3) = 14.03, *p* = 0.003] and with up to 8 years of formal education [MD = 39%, C = 7%, *X*^2^(3) = 19.88, *p* = 0.0002].

### Baseline assessments

**Figure 2:**
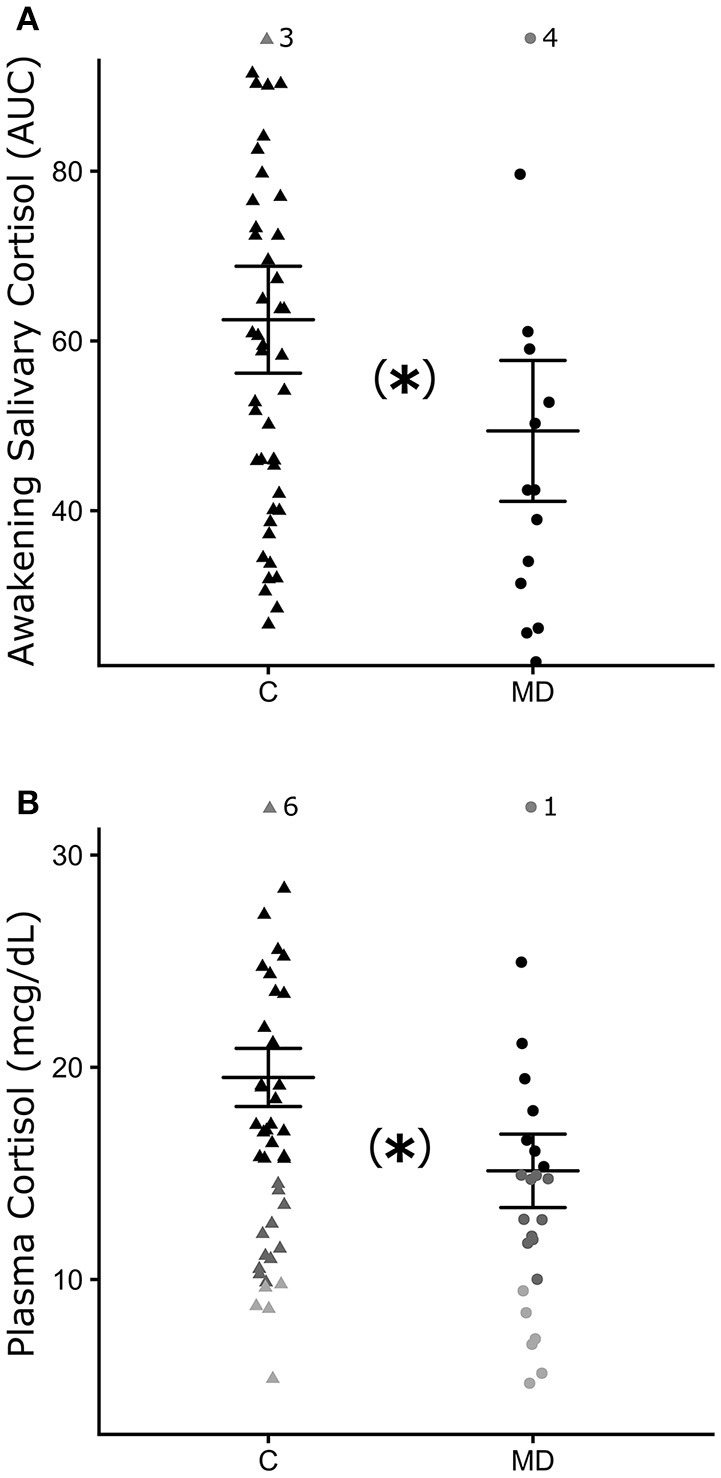
Mean and standard deviation of cortisol levels at baseline (D0). **(A)** AUC of awakening salivary cortisol for the control group (C, closed triangle) and patients (MD, closed circle). **(B)** Plasma cortisol (mcg/dL). Relative hypocortisolemia (<15 mcg/dL) = dark gray symbols and true hypocortisolemia (<10 mcg/dL) = light gray symbols). Each symbol (triangle or circle) indicate individual value of cortisol. ^*^p ≤ 0.05, statistically significant difference between-groups. GLM test and *post-hoc* Fisher.

Figure 2 shows the cortisol levels at baseline (D0). Figure 2A shows the AUC of awakening salivary cortisol response for both groups (C and MD) at baseline. AUC level at baseline was lower for patients (MD; *n* = 20, μ = 49.4 ± 8.3 cm^2^) than healthy controls (C; *n* = 41, μ = 62.5 ± 6.3 cm^2^), and these differences were independent of sex (ANCOVA main effects: Group^*^: *F* = 9.75 *df* = 1 *p* = 0.002, Sex^*^: *F* = 0.42 *df* = 1 *p* = 0.51). Figure 2B shows the results for plasma cortisol (mcg/dL). The same profile is observed in plasma cortisol at baseline, which was lower in patients (MD; *n* = 28, μ = 15.12 ± 1.73 mcg/dL) than in healthy controls (C; *n* = 43, μ = 19.52 ± 1.37 mcg/dL). Again, these differences were independent of sex (ANCOVA main effects: Group^*^: *F* = 4.71 *df* = 1 *p* = 0.03, Sex^*^: *F* = 0.89 *df* = 1 *p* = 0.34). Figure 2B also illustrates patients that show relative (n=17 and 61%) and true hypocortisolemia (*n* = 6 and 22.22%), with total plasma cortisol levels below 15 and 10 mcg/dL, respectively.

At baseline a significant positive correlation was observed between cortisol levels (plasma and AUC) in D0, for controls (*p* < 0.05 *r*_*s*_ = 0.54), but not for patients. No significant correlations were found between cortisol levels (plasma and salivary), depression severity (HAM-D and MADRS), and duration of disease for patients (Supplementary Table 1 for details).

### Acute effects of ayahuasca (dossing session)

**Figure 3:**
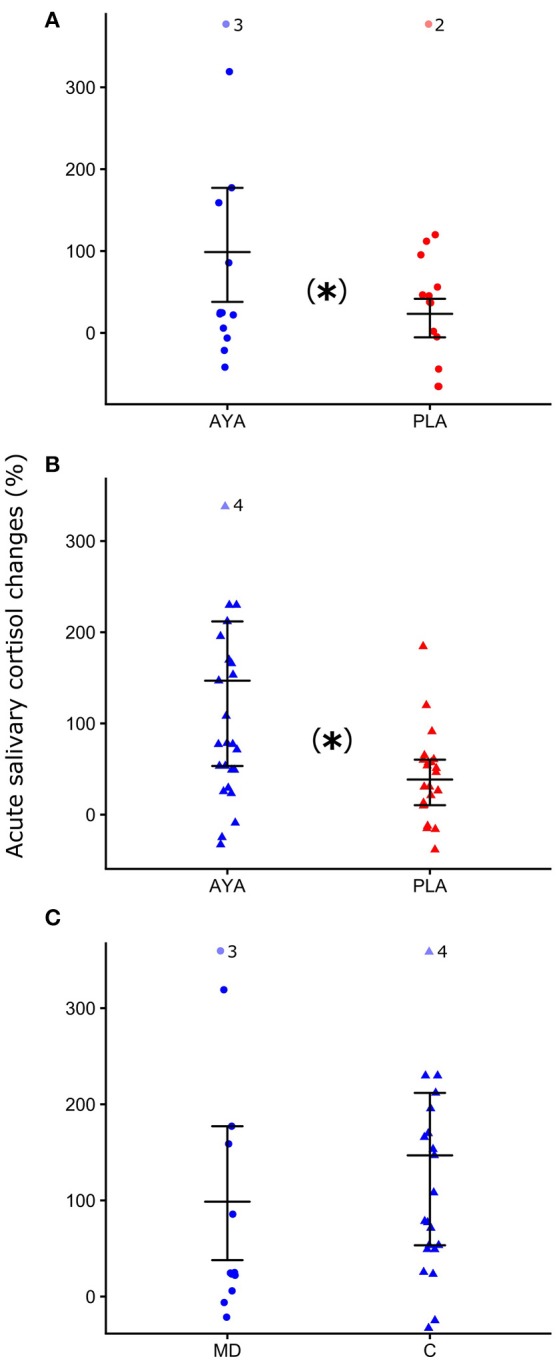
Median Q25 and Q75 of acute salivary cortisol changes (%) at 1 h 40 min into the dosing session. **(A)** For patients with major depression (MD, closed circle) after ayahuasca (AYA, blue color), or placebo (PLA, red color) ingestion. **(B)** Control group (C, closed triangle) after ayahuasca or placebo ingestion. **(C)** MD and C after ayahuasca ingestion. ^*^p ≤ 0.05, statistically significant difference between-groups. Mann-Whitney non-parametric test.

Figure 3 shows the acute salivary cortisol changes (%) during the dosing session of salivary cortisol observed 1 h 40 after ayahuasca or placebo ingestion. Figure 3A shows that patients in the ayahuasca group (*n* = 10) presented greater salivary cortisol increases (median = 98.72; Q25% = 37.89; Q75% = 177.16) compared to the placebo group (*n* = 12) (median = 23.26; Q25% = −5.44; Q75% = 41.65) (Mann-Whitney test *U* = 27 *p* = 0.03). Figure 3B shows the same analyses for the control group. Controls of the ayahuasca group (*n* = 21) showed greater increases of salivary cortisol levels (median = 146.87; Q25% = 53.32; Q75% = 211.84) compared to the placebo group (*n* = 20) (median = 38.50; Q25% = 10.33; Q75% = 60.27) (Mann-Whitney test *U* = 84 *p* = 0.01). Figure 3C compares patients and controls that ingested ayahuasca, both showing similar changes of salivary cortisol at 1 h 40 min after ingestion of ayahuasca (Mann-Whitney test *U* = 85 *p* = 0.66; patients median = 98.72; Q25% = 37.89; Q75% = 177.16, controls median = 146.87; Q25% = 53.32; Q75% = 211.84). Acute salivary cortisol changes (%) observed during dosing session for patients were not significantly correlated with MADRS scores at D2 (Supplementary Table 2 for details).

### Post-treatment assessments (D2)

**Figure 4:**
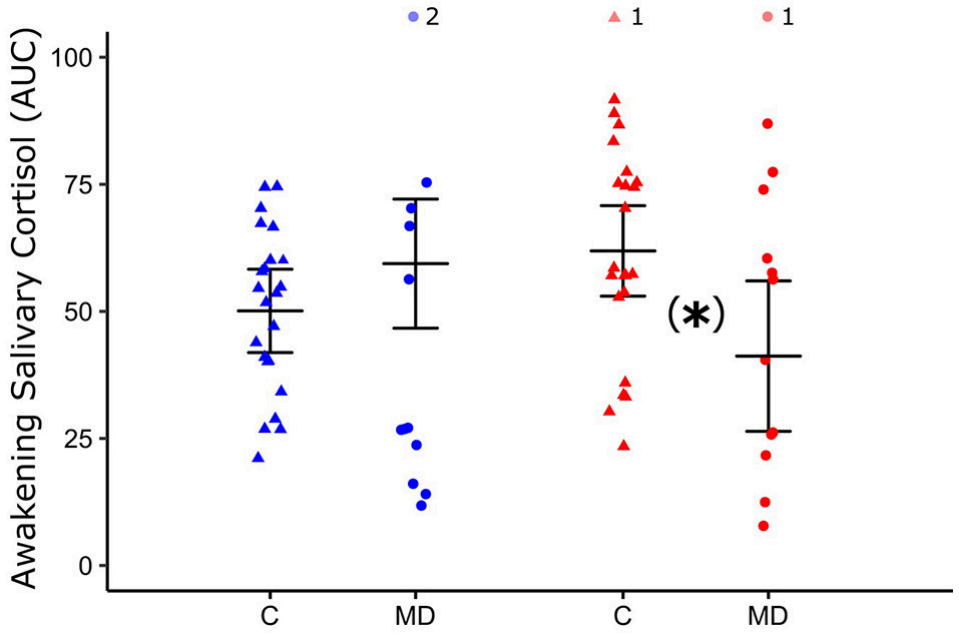
Mean and standard deviation of area under the curve (AUC) of awakening salivary cortisol 48 h after dosing (D2) of control group (C, closed triangle) and patients with major depression (MD, closed circle) who ingested ayahuasca (blue color) or placebo (red color). ^*^p < 0.05, statistically significant difference between-groups. GLM test and *post-hoc* Fisher.

No changes in AUC between D0 and D2, for within groups and treatments were observed. (GLM: Group^*^Treatment^*^Days: *F* = 4.57, *p* = 0.03, Supplementary Tables 3, 4 for details). At D2, AUC of patients who ingested ayahuasca (μ = 59.4 ± 12.7 cm^2^) is similar to values of controls group who ingested ayahuasca (μ = 50.1 ± 8.2 cm^2^) (Fisher *post-hoc*: *p* = 0.45). On the other hand, patients that ingested placebo continued presenting lower AUC (μ = 41.2 ± 14.8 cm^2^) than controls that ingested placebo (μ = 61.9 ± 8.9 cm^2^) (Fisher *post-hoc*: *p* = 0.03). Influence of sex was not observed for AUC (supplementary Table 3 for details). Figure 4 shows AUC of awakening salivary cortisol response for both groups (C and MD) and treatments (ayahuasca and placebo) 48 h after dosing session (D2). Individual changes of AUC between D0 and D2 were illustrated for each group and treatment in Supplementary Figure 1.

For plasma cortisol no significant changes were observed between D0 and D2, within both groups (MD and C) and treatment (ayahuasca or placebo) (GLM: Group^*^Treatment^*^Days: *F* = 1.24, *p* = 0.27, Supplementary Table 5 for details). Individual changes in PC between D0 and D2 are illustrated for each group and treatment in Supplementary Figure 2.

No statically significant correlations were observed between plasma cortisol and AUC of awakening salivary cortisol of D2 for patients nor controls, nor with MADRS scores in the patients group. A detailed description of the *Spearman* correlation results are presented in Supplementary Table 2.

## Discussion

In this study we found basal hypocortisolemia and blunted awakening salivary cortisol response in patients with treatment-resistant depression, compared to healthy controls. During dosing, 1 h 40 min after ingestion, we observed significant acute increases in salivary cortisol in the ayahuasca groups (C and MD), compared to placebo groups (C and MD). Moreover, 48 h after the dosing session (D2) with ayahuasca, patients' awakening salivary cortisol response is similar to levels detected in healthy controls. This was not observed in patients that ingested placebo, which continued showing different AUC of awakening salivary cortisol response compared to the control group. No significant differences were found in D2 regarding plasma cortisol.

In our recent randomized placebo-controlled trial we observed a significant antidepressant effect after a single dosing session with ayahuasca, when compared to placebo. Depression severity diminished significantly 1 day after dosing, and between-group differences increased from D1 to D7 (43).

Our results partially corroborate our initial hypothesis. We expected different levels of cortisol between patients and controls at baseline and, in fact, cortisol levels (both plasma and salivary) were reduced in the group of patients when compared to healthy controls. We also estimated that the cortisol levels will be correlated with the severity and/or duration of disease. Unexpectedly, cortisol levels (plasma and salivary) observed in the group of patients were not correlated with severity nor disease duration. Nevertheless, at baseline, a positive correlation between plasma and AUC of awakening salivary cortisol levels was found for controls, but not for patients.

Cortisol is a steroid hormone that triggers stress response in an adaptive way: it increases cardiovascular and respiratory activity, mobilizes glucose to provide enough fuel to remove the stressor and limit acute inflammation processes (45). Not only the excess, but also the reduction of cortisol is believed to be harmful. Major depression has been traditionally associated with hypercortisolism, but an increasing number of studies have found evidence of hypocortisolism in depression (8, 46, 47). Chronic decreased cortisol levels induce non-specific symptoms such as general malaise, weakness, low blood pressure, muscle weakness, loss of appetite and weight, gastrointestinal complaints and immunological dysfunction (48), some of which are very common in major depression. Besides depression, low cortisol levels have been found in some other conditions, such as Addison's disease (49), adrenal insufficiency (50, 51), and post-traumatic stress disorders (52).

In the literature, more severe depression, marked by chronic or recurrent disease episodes, is frequently accompanied by hypocortisolemia (8, 46, 47). These patients often exhibit long-term exposition to stressors, which in the early phase of the disease may induce chronic upregulated activity of the HPA axis and hypercortisolemia, followed by reducing cortisol to very low pathological levels (53, 54). In addition, evidence suggests that prolonged use of some antidepressants may also lead to increased expression of cortisol receptors and increased sensitivity of negative feedback, thus decreasing cortisol levels to under homeostatic values (55). Our patients were all treatment-resistant and used an average of 3.86 ± 1.66 different types of antidepressants, which could in turns induced hypocortisolemia. Low cortisol levels in depression have also been associated with maladaptive coping style (56) and unfavorable socioeconomic status (13, 57). Patients in this study had a particular profile: they were all from low socioeconomic status background, low educated and living in a low-income household, in a stressful environment. Previous studies have reported similar results. They found low levels of cortisol at awakening in patients with depression from the same region, compared to healthy controls (58), and with patients from Canada (58). Due to significant levels of poverty and scarce government investments in this region, this population usually is submitted to a long exposure of adverse events in life, and early in life they cope with precarious physical and physiological health, as well as with cumulative social and economic disadvantageous conditions.

The etiology of hypocortisolism is explained by various theories. One of them shows that a greater and decompensated sensitivity in negative feedback of the HPA axis deregulates cortisol secretion (54). Also, hypocortisolism is associate to adrenal insufficiency, a failure to produce cortisol and a decrease or increase in Adrenocorticotrophic hormone (ACTH) concentrations that depend on the type of failure, whether primary or secondary, respectively (48). New evidence also points to the participation of paracrine and autocrine messengers in adrenal failure (59). While several theories try to explain the etiology of hypocortisolism, it seems that the best approach to elucidate this pathophysiological process involves the integration of all these theories.

The regulation of cortisol is an important physiological aspect on the way of achieving biological health, since cortisol is an integrative hormone with large potential of body modulation, particularly involved in the etiology of depression, engaging the immune, and monoaminergic systems (53, 60, 61). Moreover, optimal levels of cortisol are necessary to induce neurogenesis, possibly due to its modulatory properties over brain-derived neurotrophic factor (BDNF) (62, 63). This relation seems to be an important factor in the etiology of depression, considering that the effectiveness of traditional antidepressants seems to be mediated by neuronal plasticity and neurogenesis (64).

The reference values for the diagnosis of corticosteroid insufficiency vary. Some studies define hypocortisolemia when cortisol levels are below 15 mcg/dL, others define it under 10 mcg/dL (65–67). Studies that evaluated the utility of basal morning serum cortisol measurements in the diagnosis of adrenal insufficiency showed that a threshold of 10 mcg/dL leads to 77% of specificity, and 62% sensitivity, defined by a subnormal serum cortisol response to insulin-induced hypoglycemia (68, 69). Therefore, in this study we considered 10 mcg/dL as cutoff reference value for true hypocortisolemia. We observed that 61% of our patients showed relative hypocortisolemia (<15 mcg/dL), and 22% true hypocortisolemia (<10 mcg/dL). On the other hand, awakening salivary cortisol response has been less used than plasma cortisol to monitor adrenal insufficiency, as it has not been fully validated as a diagnostic test.

Plasma cortisol levels were positively correlated with saliva cortisol levels at baseline for controls, but not for patients, in line with previous studies that observed a correlation between total plasma and salivary cortisol in healthy subjects (70). The absence of correlation in the groups of patients could be due to a malfunction of the HPA axis (70) or by changes in concentration of CBG (Cortisol Binding Globulin), its transporter protein in the plasma (71).

Moreover, we also supposed that ayahuasca, but not placebo, could acutely increase cortisol levels, after the dosing session, in patients and controls. The large increase in cortisol levels by ayahuasca found 1 h 40 after intake, for health and depressive volunteers, corroborated our hypothesis. Previous studies in healthy subjects have also reported increased cortisol levels during the acute effects of ayahuasca (72–74), N,N-DMT (75), psilocybin (76, 77), and Lysergic Acid Diethylamide (LSD) (78). One should bear in mind that our patients presented, in general, hypocortisolemia, and blunted awakening salivary cortisol response. It is reasonable to consider that subjects who took ayahuasca, which immediately increased cortisol levels, probably were acutely benefited by the ingestion of the ayahuasca, thereby leading to a direction of achieving hormonal homeostasis.

The modulation of the HPA axis by antidepressants depends on the type of antidepressant used and treatment duration, acute or chronic. Noradrenaline or serotonin (5HT) reuptake-inhibiting antidepressants, such as reboxetine and citalopram, acutely stimulate cortisol secretion in healthy volunteers, probably due the elevation in 5HT levels (79, 80). On the other hand, some antidepressants, as mirtazapine, acutely inhibits cortisol release, probably due to its selective antagonism at 5-HT_2_ receptors (79). It is interesting to notice that the long-term effects of antidepressants are frequently opposite to the acute ones. In the long way, reboxetine up-regulates cortisol receptors function, repairs the disturbed feedback control and normalizes HPA axis. Mirtazapine, within 1 week, markedly reduces HPA axis activity in depressed patients (79, 80). If the patient is resistant to treatments, and uses antidepressants by years, the long-term effects could be disturbed and followed by an unfavorable physiological response, and as cited above, the chronic use of some antidepressant could induce hypocortisolemia.

Here, the acute increases of cortisol levels by the ayahuasca can be due the rise in serotonin induced by the N,N-DMT, and β-carbolinic alkaloids (31), likewise, other studies support the modulation by serotonin on the secretion of Corticotrophin Releasing Hormone (CRH) and ACTH both at the hypothalamic and pituitary glands (81).

After 48 h of the dosing session (D2), no changes in cortisol levels were observed comparing to baseline levels, within each group, and between treatments, neither in AUC of awakening salivary cortisol response nor in the plasma. These results were different than the claim of our initial hypothesis: previously, we expected an increase in cortisol levels (plasma and salivary) in D2. Individual analysis of AUC and PC at D0 and D2 showed a large individual variability. Some studies also faced with this large individual variability at baseline levels and response of cortisol (82). However, after 48 h from the dosing session, the AUC of awakening salivary cortisol response of patients that ingested ayahuasca, and not placebo, is similar to control that ingested ayahuasca. This similarity of AUC between controls and patients that were treated with ayahuasca points to a beneficial physiological modulation by ayahuasca on awakening salivary cortisol response. This finding probably corroborates with the improvement of several physiological systems, emotional and cognitive aspects that are regulated by cortisol (83, 84). Despite cortisol levels showed large inter and intra-individual variability, and as the area under the curve of awakening salivary cortisol consisted of three saliva samples collected in the early morning hours, some studies have been considering the awakening salivary cortisol response as more reliable biomarker than plasma. Awakening salivary cortisol response was demonstrated to be sensible to treatment by antidepressants (47, 85–88), probably because this index is less modulated by circadian clock and daily stressors than plasma cortisol levels (89).

Some studies with animal models of depression, rodents and non-human primates, observed positive antidepressant effects with the use of ayahuasca or its specific components (90–92). Using the recently validated translational animal model of depression (93), young marmosets (*Callithrix jacchus*) that showed hypocortisolemia, were treated with nortriptyline during 7 days, a tricyclic antidepressant, or with a single dose of ayahuasca. It was observed that ayahuasca increased fecal cortisol levels until 48 h after it ingestion, and presented more notable antidepressant effects than nortriptyline, since it reverted depressive-like behaviors and regulated cortisol levels faster and longer, when compared with nortriptyline (93).

Although there is great evidence that normalization of HPA axis function is essential for the successful treatment of depressive patients (89, 94, 95) differently of our hypothesis, herein changes in cortisol levels induced by ayahuasca were not correlated with improvement in depressive symptoms observed from D1 to D7 after it intake. We speculate that this fact is probably due to the high heterogeneity of patients and relative small sample of volunteers per group in this study.

The present study supports the involvement of the HPA axis in the physiopathology of depression and the modulation of salivary cortisol by a single session with ayahuasca, both in depressive patients and healthy volunteers. The cortisol acts in regulation of distinct physiological, cognitive and emotional pathways, and previous studies suggested that regulation of its levels to standard values is considered as an important part of depression treatment. Thus, we argue that ayahuasca should be further investigated as treatment of depression. Taking these findings in account, this work contributes significantly to support the return of clinical studies with natural psychedelics applied to mental disorders.

## Author contributions

NG-C, BL-S, DdA, JM-d-O, EA, and FP-F designed the experiments; RdA and AG measured hormonal data; FF, FP-F, HO, AG, and ES collected experimental data, carried out statistical analysis and prepared the figures; NG-C, BL-S, DdA, FP-F, AG, and ES: prepared the manuscript.

### Conflict of interest statement

The authors declare that the research was conducted in the absence of any commercial or financial relationships that could be construed as a potential conflict of interest.
