# Supplementary material for: Cortisol Modulation by Ayahuasca in Patients With Treatment Resistant Depression and Healthy Controls
Source: Front Psychiatry. 2018 May 8;9:185. doi: 10.3389/fpsyt.2018.00185 (PMC5952178; doi:10.3389/fpsyt.2018.00185)
Supplement: Supplementary file 1 [file Table_1.pdf]

Table 1. Statistical values of *Spearman* correlations test in baseline between plasma cortisol, area under the curve (AUC) of awakening salivary cortisol response of patients and controls, scores of scales of depression (HAM-D and MADRS) and duration of disease of patients.

**Variables**	**PC ^{C}**	**PC ^{MD}**	**AUC ^{C}**	**AUC ^{MD}**	**DD**
**PC ^{C}**	1,000000	0,256705	0,543311	-0,108059	0,012386
**PC ^{MD}**	0,256705	1,000000	0,039316	0,072650	-0,012669
**AUC ^{C}**	0,543311	0,039316	1,000000	-0,246923	0,023605
**AUC ^{MD}**	-0,108059	0,072650	-0,246923	1,000000	0,101432
**MADRS**	-0,150254	-0,314476	0,216707	-0,236558	0,144029
**HAM-D**	-0,197773	-0,211909	-0,188226	-0,071365	0,174227
**DD**	0,012386	-0,012669	0,023605	0,101432	1,000000

PC: Plasma Cortisol; AUC: Area Under the Curve; C: Control group; MD: Patients with Major Depression; MADRS: Montgomery-Åsberg Depression Rating Scale; HAM-D: Hamilton Depression Rating Scale; DD: Duration of disease. All values in black correspond to statistical significance and values in gray to non-significant ones.
