# Supplementary material for: Cortisol Modulation by Ayahuasca in Patients With Treatment Resistant Depression and Healthy Controls
Source: Front Psychiatry. 2018 May 8;9:185. doi: 10.3389/fpsyt.2018.00185 (PMC5952178; doi:10.3389/fpsyt.2018.00185)
Supplement: Supplementary file 2 [file Table_2.pdf]

Table 2. Statistical values of *Spearman* correlations test between acute salivary cortisol changes (%), plasma cortisol and AUC of awakening salivary cortisol in D2, for patients and controls of each treatment, and scores of MADRS for patients of both treatments.

| Variables | % C AYA | % C PLA | % MD AYA | % MD PLA | PC C AYA | PC C PLA | PC MD AYA | PC MD PLA | AUC C AYA | AUC C PLA | AUC MD AYA | AUC MD PLA |
|---|---|---|---|---|---|---|---|---|---|---|---|---|
| **% C AYA** | 1,000000 | 0,210526 | 0,030303 | -0,075000 | -0,509091 | 0,261039 | -0,345055 | 0,432967 | 0,272180 | 0,192982 | -0,146853 | 0,244755 |
| **% C PLA** | 0,210526 | 1,000000 | 0,030303 | -0,150000 | -0,167532 | 0,225296 | -0,441758 | -0,213187 | 0,445113 | 0,166234 | 0,041958 | 0,020979 |
| **% MD AYA** | 0,030303 | 0,030303 | 1,000000 | 0,066667 | -0,212121 | -0,212121 | 0,006061 | -0,283333 | 0,163636 | 0,050000 | 0,283333 | -0,547619 |
| **% MD PLA** | -0,075000 | -0,150000 | 0,066667 | 1,000000 | -0,110714 | 0,139286 | 0,362637 | -0,076923 | 0,090110 | -0,239560 | 0,426573 | 0,055944 |
| **PC C AYA** | -0,509091 | -0,167532 | -0,212121 | -0,110714 | 1,000000 | 0,278374 | 0,173626 | -0,305495 | -0,333766 | -0,066165 | 0,160839 | -0,111888 |
| **PC C PLA** | 0,261039 | 0,225296 | -0,212121 | 0,139286 | 0,278374 | 1,000000 | -0,437363 | 0,178022 | -0,070130 | 0,412987 | -0,104895 | 0,020979 |
| **PC MD AYA** | -0,345055 | -0,441758 | 0,006061 | 0,362637 | 0,173626 | -0,437363 | 1,000000 | -0,230769 | -0,170330 | -0,434066 | 0,342657 | 0,154545 |
| **PC MD PLA** | 0,432967 | -0,213187 | -0,283333 | -0,076923 | -0,305495 | 0,178022 | -0,230769 | 1,000000 | -0,104396 | 0,302198 | -0,063636 | 0,594406 |
| **AUC C AYA** | 0,272180 | 0,445113 | 0,163636 | 0,090110 | -0,333766 | -0,070130 | -0,170330 | -0,104396 | 1,000000 | 0,268421 | 0,381818 | -0,154545 |
| **AUC C PLA** | 0,192982 | 0,166234 | 0,050000 | -0,239560 | -0,066165 | 0,412987 | -0,434066 | 0,302198 | 0,268421 | 1,000000 | -0,354545 | 0,109091 |
| **AUC MD AYA** | -0,146853 | 0,041958 | 0,283333 | 0,426573 | 0,160839 | -0,104895 | 0,342657 | -0,063636 | 0,381818 | -0,354545 | 1,000000 | 0,516667 |
| **AUC MD PLA** | 0,244755 | 0,020979 | -0,547619 | 0,055944 | -0,111888 | 0,020979 | 0,154545 | 0,594406 | -0,154545 | 0,109091 | 0,516667 | 1,000000 |
| **MADRS D2 AYA** | -0,147253 | 0,076923 | -0,503030 | 0,085714 | 0,221978 | 0,283516 | 0,287912 | -0,401099 | 0,049451 | 0,027473 | -0,209790 | -0,118182 |
| **MADRS D2 PLA** | 0,167813 | 0,409904 | -0,690476 | -0,316369 | 0,079780 | 0,063274 | -0,339755 | 0,171629 | 0,133100 | 0,368639 | 0,030303 | 0,393939 |

%: Acute salivary cortisol change; C: Control group; AYA: Ayahuasca; PLA: Placebo; MD: Patients with Major Depression; PC: Plasma Cortisol; AUC: Area Under the Curve of awakening salivary cortisol; MADRS: Montgomery-Åsberg Depression Rating Scale; D0: Baseline; D2: 48h after dosing session. All values in black correspond to statistical significance and values in gray to non-significant ones.
