# Supplementary material for: Cortisol Modulation by Ayahuasca in Patients With Treatment Resistant Depression and Healthy Controls
Source: Front Psychiatry. 2018 May 8;9:185. doi: 10.3389/fpsyt.2018.00185 (PMC5952178; doi:10.3389/fpsyt.2018.00185)
Supplement: Supplementary file 3 [file Table_3.pdf]

Table 3. Statistical values of main effects and interaction of General Linear Models (GLM) used for analyze of the changes of AUC (area under the curve) of awakening salivary cortisol between baseline (D0) and 48h after dosing session (D2), for control group and patients with major depression of both treatments (ayahuasca and placebo).

EFFECT	F	p	DF
**INTERCEPT**	4890,443	0,000000	1
**GROUP**	3,776	0,057310	1
**TREATMENT**	0,198	0,658464	1
**SEX**	0,294	0,589763	1
**GROUP*TREATMENT**	0,009	0,923246	1
**GROUP*SEX**	1,842	0,180482	1
**TREATMENT*SEX**	0,300	0,586259	1
**GROUP*TREATMENT*SEX**	1,956	0,167731	1
**ERROR**			53
**DAYS**	2,604	0,112554	1
**DAYS*GROUP**	0,653	0,422796	1
**DAYS*TREATMENT**	0,963	0,330852	1
**DAYS*SEX**	2,515	0,118748	1
**DAYS*GROUP*TREATMENT**	4,578	0,037010	1
**DAYS*GROUP*SEX**	2,793	0,100559	1
**DAYS*TREATMENT*SEX**	2,640	0,110128	1
**DAYS*GROUP*TREATMENT*SEX**	0,477	0,492980	1
**ERROR**			53

DF: Degree of freedom; Treatment (ayahuasca and placebo); Group (control and patients with major depression); Days (D0 and D2); Sex (man and woman) . All values in black correspond to statistical significance and values in gray to non-significant ones.
