# Supplementary material for: Cortisol Modulation by Ayahuasca in Patients With Treatment Resistant Depression and Healthy Controls
Source: Front Psychiatry. 2018 May 8;9:185. doi: 10.3389/fpsyt.2018.00185 (PMC5952178; doi:10.3389/fpsyt.2018.00185)
Supplement: Supplementary file 4 [file Table_4.pdf]

Table 4. Statistical values of Fisher *post-hoc* test used for analyzed the changes of AUC (area under the curve) of awakening salivary cortisol between baseline (D0) and 48h after dosing session (D2) for control group and patients with major depression of both treatments (ayahuasca and placebo).

GROUP	TREATMENT	DAYS	DM AYA AUC D0	DM AYA AUC D2	DM PLA AUC D0	DM PLA AUC D2	C AYA AUC D0	C AYA AUC D2	C PLA AUC D0	C PLA AUC D2
**MD**	AYA	AUC D0		0,569491	0,940631	0,555672	0,178394	0,198591	0,118311	0,026126
**MD**	AYA	AUC D2	0,569491		0,725552	0,531234	0,130499	0,457654	0,070826	0,144383
**MD**	PLA	AUC D0	0,940631	0,725552		0,291964	0,165489	0,174705	0,110449	0,023511
**M**	PLA	AUC D2	0,555672	0,531234	0,291964		0,017246	0,155320	0,008237	0,036840
**C**	AYA	AUC D0	0,178394	0,130499	0,165489	0,017246		0,163748	0,767919	0,566955
**C**	AYA	AUC D2	0,198591	0,457654	0,174705	0,155320	0,163748		0,311904	0,375738
**C**	PLA	AUC D0	0,118311	0,070826	0,110449	0,008237	0,767919	0,311904		0,702115
**C**	PLA	AUC D2	0,026126	0,144383	0,023511	0,036840	0,566955	0,375738	0,702115	

MD: Patients with Major Depression; C: Control group; AYA: Ayahuasca; PLA: Placebo; AUC: Area Under the Curve of awakening salivary cortisol; D0: Baseline; D2: 48h after dosing session. All values in black correspond to statistical significance and values in gray to non-significant ones.
