# Supplementary figures and images for: Cortisol Modulation by Ayahuasca in Patients With Treatment Resistant Depression and Healthy Controls
Source: Front Psychiatry. 2018 May 8;9:185. doi: 10.3389/fpsyt.2018.00185 (PMC5952178; doi:10.3389/fpsyt.2018.00185)

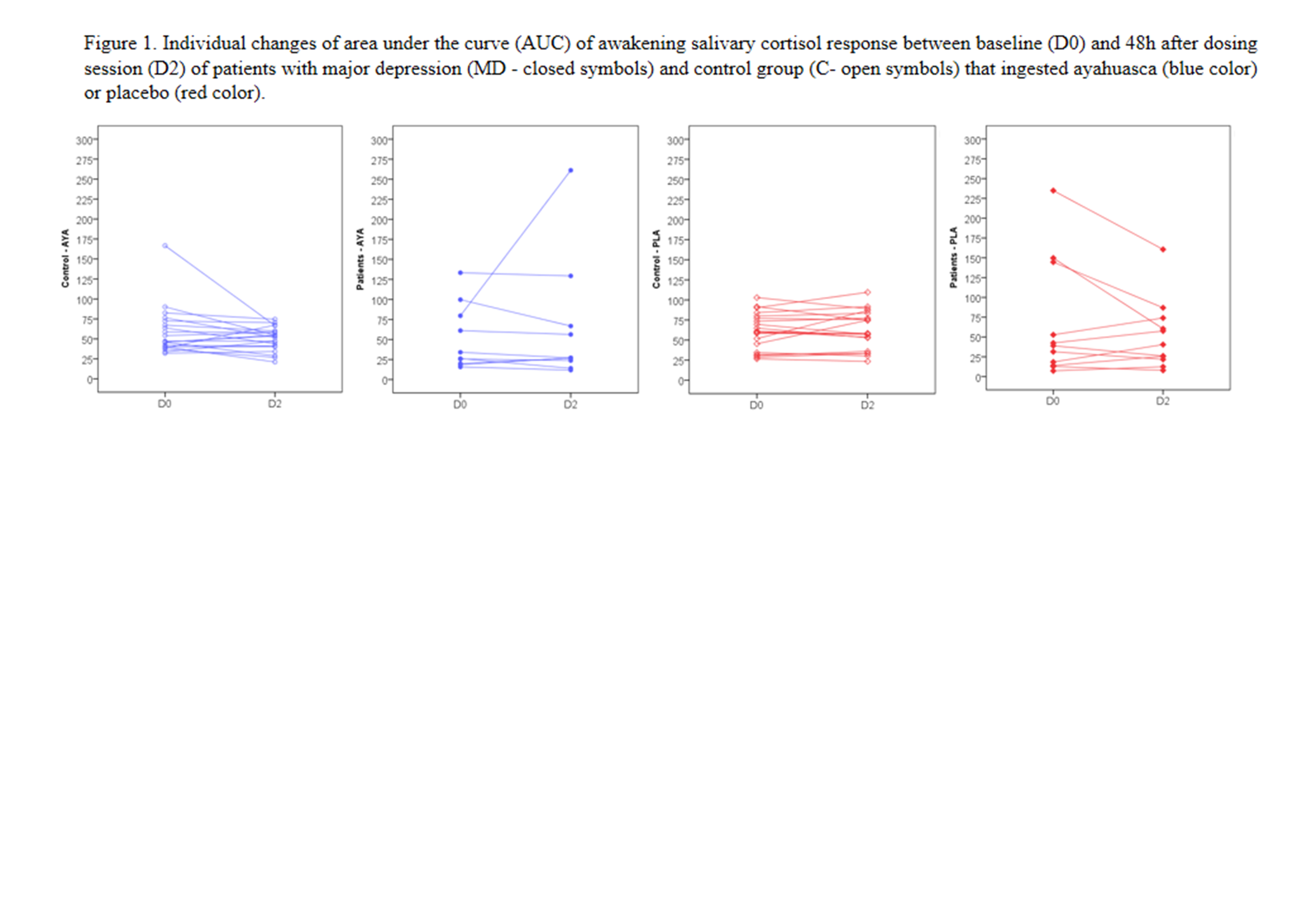

Supplement: Supplementary file 6 [file Image_1.TIF]

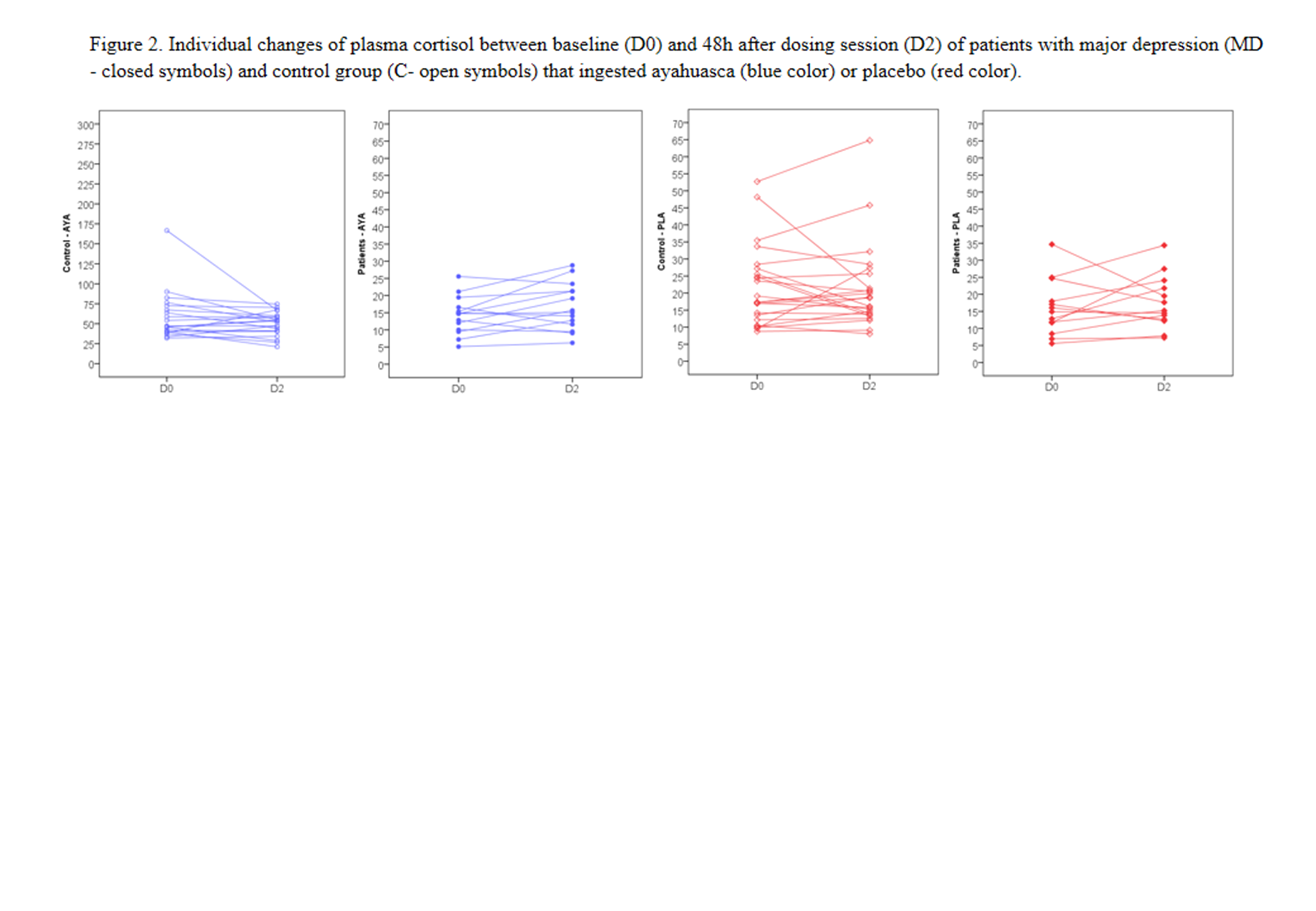

Supplement: Supplementary file 7 [file Image_2.TIF]
